# Artificial Intelligence-Based Evaluation of Oral Potentially Malignant Disorders Using Photographic and Clinical Data: A Retrospective Exploratory Diagnostic Accuracy Study

**DOI:** 10.3390/dj14100657

**Published:** 2026-10-10

**Authors:** Domenico De Falco, Fabiana Pinto, Alberta Lucchese, Massimo Petruzzi

**Affiliations:** 1 Interdisciplinary Department of Medicine, University of Bari “Aldo Moro”, 70124 Bari, Italy; 2 Multidisciplinary Department of Medical-Surgical and Dental Specialties, University of Campania “Luigi Vanvitelli”, 80138 Naples, Italy

**Keywords:** oral medicine, oral pathology, oral potentially malignant disorders, artificial intelligence

## Abstract

**Objective:** The objective of this study was to evaluate the diagnostic performance of GPT-5.2 Thinking in identifying oral potentially malignant disorders within a patient sample that also included oral carcinomas and other oral cavity lesions, using intraoral photographs and associated clinical data. **Methods**: This retrospective exploratory diagnostic accuracy study included 103 oral lesions: 69 oral potentially malignant disorders, 25 oral carcinomas, and 9 other oral pathologies. Each case was assessed in three independent sessions, and the majority diagnosis was compared with the final clinical and/or histopathological reference diagnosis. **Results**: For oral potentially malignant disorders, sensitivity was 92.75%, specificity was 88.24%, accuracy was 91.26%, positive predictive value was 94.12%, negative predictive value was 85.71%, and Cohen’s kappa was 0.804. Sensitivity was 81.82% for homogeneous leukoplakia, 78.57% for non-homogeneous leukoplakia, and 86.36% for oral lichen planus. For oral carcinomas, sensitivity was 84.00%, specificity was 97.44%, accuracy was 94.17%, positive predictive value was 91.30%, negative predictive value was 95.00%, and Cohen’s kappa was 0.837. **Conclusions:** GPT-5.2 Thinking showed promising diagnostic performance in this exploratory case-enriched dataset, but predictive values require validation in representative clinical populations.

## 1. Introduction

Artificial intelligence (AI) is assuming an increasingly prominent role across multiple scientific disciplines, with particularly significant applications in medicine [1]. In the clinical setting, AI has the potential to enhance the quality of patient care by supporting diagnostic accuracy and optimizing therapeutic decision-making, thereby contributing to the reduction in human error and improving overall healthcare outcomes [1,2]. Machine learning and neural network technologies can emulate certain functions of the human brain and facilitate rapid learning [3]. In medicine, this process can occur in several ways, for instance, through the use of clinical cases accompanied by photographic documentation and patient medical histories [3]. For this reason, access to large and comprehensive datasets is essential for the training of AI systems [4,5]. Another key factor in reducing diagnostic error is the careful definition, through prompts, of the boundaries within which AI can be expected to perform diagnostically. These boundaries are often determined by established diagnostic criteria and official disease classification systems [6,7]. Oral potentially malignant disorders (OPMDs) comprise a heterogeneous group of lesions associated with a variable risk of progression to oral malignancy, ranging from low to high [8]. The overall average rate of malignant transformation is estimated to be approximately 8% [9]. Even today, these disorders are frequently underdiagnosed, diagnosed at a late stage, or inadequately monitored over time [8,9]. This may occur for several reasons, including the limited experience of dentists and general practitioners in recognizing and assessing such lesions [10]. Delayed diagnosis often results in an unfavorable prognosis and, in cases of malignant transformation, is associated with lower 5-year survival rates [10,11,12]. In this context, AI could enhance both the accuracy and the efficiency of early diagnosis and prognostic assessment in OPMDs [13]. First, by accessing electronic health records, AI models may help identify population subgroups at increased risk. They may also support pathologists by improving the detection and segmentation of histological slides, thereby highlighting areas with a greater likelihood of disease progression. In addition, AI can be used to identify biomarkers following cytological and salivary testing [14,15]. The most promising application of AI in oral medicine concerns support in clinical diagnosis. AI-assisted diagnosis could play a key role in reducing the number of underdiagnosed cases and improving the early detection of these conditions [13]. AI may be applied through the photographic analysis of clinical lesions, as well as through interpretive support in the assessment of vital staining patterns or tissue autofluorescence loss [13,14,16]. A range of AI software tools is currently available, some designed for specific medical or scientific disciplines and others intended for more general use [17]. Several studies have been conducted in oral medicine to evaluate the diagnostic potential of AI through photographic analysis. Specifically, in the field of OPMDs, the literature on AI applied to clinical photographs is still predominantly based on image data alone; models that systematically integrate clinical and medical history information are, to date, few, preliminary, and not yet sufficiently validated in prospective and multicenter settings [18,19,20]. The aim of this study was to evaluate the diagnostic performance of a general-purpose AI software program (GPT-5.2 Thinking) in identifying OPMDs within a sample of oral lesions through the analysis of photographic images and associated clinical data. A secondary objective was to assess the diagnostic sensitivity of the AI system across the different OPMDs subcategories. Finally, a further objective was to evaluate the diagnostic sensitivity of the AI system in identifying oral carcinomas within the overall patient sample.

## 2. Materials and Methods

### 2.1. Study Design and Setting

This Retrospective Exploratory Diagnostic Accuracy Study was conducted between December 2025 and April 2026 at the Unit of Oral Medicine and Oral Pathology, University of Bari, Italy, in accordance with the World Medical Association Declaration of Helsinki, as revised in 2024 [21]. Ethical approval was obtained from the Independent Ethics Committee of the Azienda Ospedaliero-Universitaria Consorziale Policlinico di Bari (authorization no. 7268). The study was reported in compliance with the STARD-AI guidelines for diagnostic accuracy studies using AI [22] (Supplementary Table S1).

### 2.2. Study Sample and Eligibility Criteria

**Figure 1:**
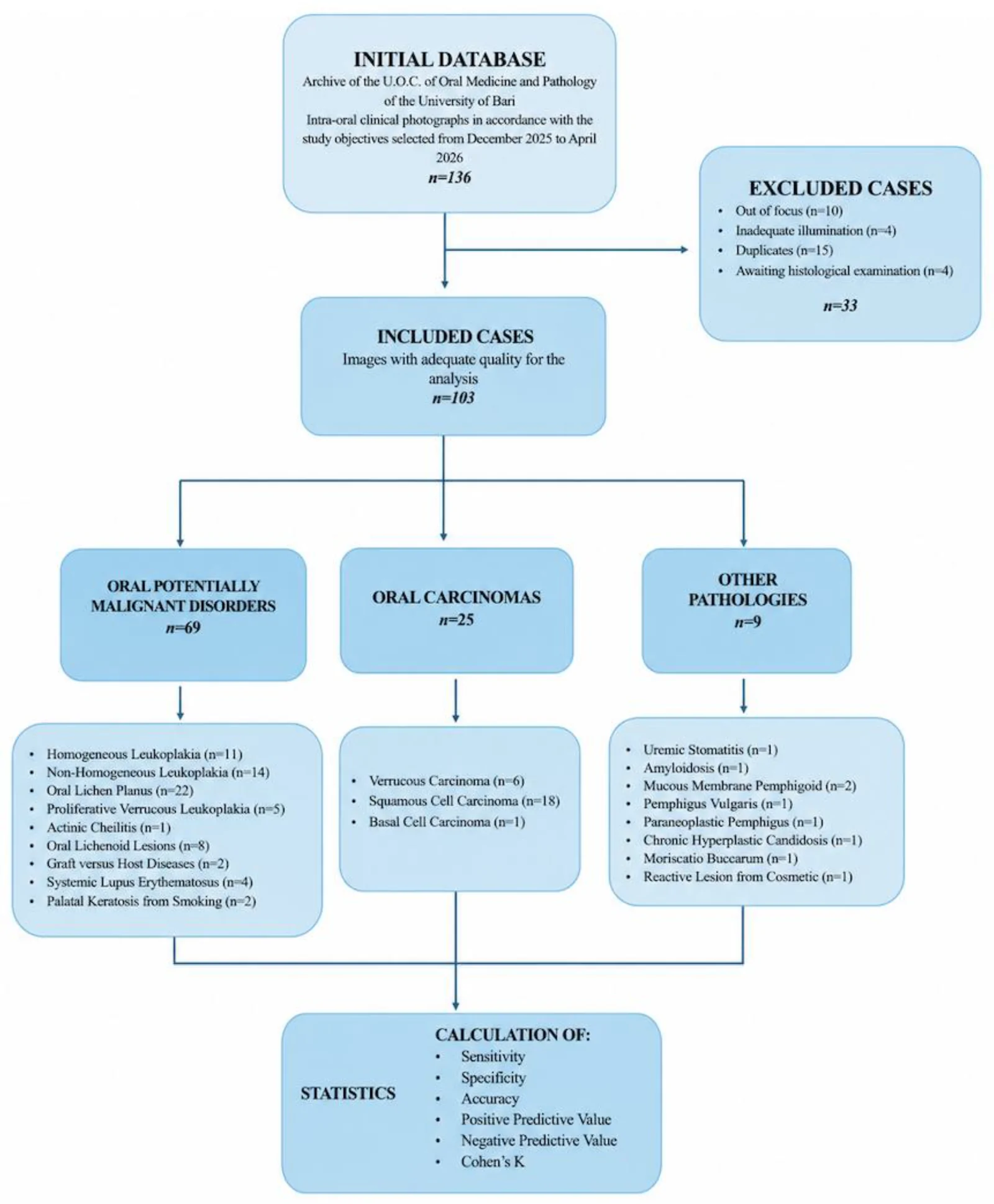
Flow diagram of the selection of intraoral clinical photographs and their distribution according to diagnostic category.

The study was based on the analysis of 103 clinical photographs (103 patients) of oral lesions acquired using a SONY digital camera (ILCE-7M3; Sony Corporation, Tokyo, Japan). The 103 selected photographs were obtained from an initial sample of 136 clinical intraoral photographs. These images were selected based on the quality of the photographs. Blurred or out-of-focus images (*n* = 10), as well as overexposed or underexposed photographs (*n* = 4) that did not accurately reproduce the clinical chromatic pattern of the lesion, were excluded. Duplicate images (*n* = 15) were also excluded unless they were clearly identifiable as part of a serial follow-up. Finally, photographs for which pathological examination results were still pending were also excluded (*n* = 4) (Figure 1).

All photographs included in the analysis had been obtained before pathological sampling or at the time patients presented with acute symptoms. All enrolled patients had a definitive clinical and/or pathological diagnosis. Because the primary objective of the study was to evaluate the ability of the AI system to identify OPMDs, a deliberately case-enriched sample with a higher proportion of OPMDs was selected to ensure adequate representation of the target condition and to permit a clinically meaningful exploratory assessment of diagnostic sensitivity. The resulting diagnostic distribution was therefore not intended to reflect disease prevalence in the general population or in primary dental care.

The image dataset comprised 103 oral lesions, including 69 OPMDs, 25 oral carcinomas (OCs), and 9 other oral pathologies. The OPMD group consisted of 11 cases of homogeneous oral leucoplakia (HOL), 14 cases of non-homogeneous oral leucoplakia (NHOL), 22 cases of oral lichen planus (OLP), 5 cases of proliferative verrucous leucoplakia (PVL), 1 case of actinic cheilitis (AC), 8 cases of oral lichenoid lesions (OLLs), 2 cases of graft-versus-host disease (GVHD), 4 cases of systemic lupus erythematosus (SLE), and 2 cases of palate keratosis associated with smoking (PKAS). The OC group included 6 cases of oral verrucous carcinomas (OVCs), 18 cases of oral squamous cell carcinomas (OSCCs), and 1 case of basal cell carcinoma (BCC). The remaining oral pathologies comprised 1 case of uremic stomatitis (US), 1 case of amyloidosis, 2 cases of mucous membrane pemphigoid (MMP), 1 case of pemphigus vulgaris (PV), 1 case of paraneoplastic pemphigus (PNP), 1 case of chronic hyperplastic candidiasis (CHC), 1 case of morsicatio buccarum (MB), and 1 cosmetic reaction lesion (CRL). For each case, the reference diagnosis was established according to accepted diagnostic criteria for the specific condition. Histopathological confirmation was used when required for diagnosis, whereas expert clinical assessment was used for conditions for which established criteria permit a clinical diagnosis without biopsy.

### 2.3. Image Anonymization and Standardization

All images were anonymized by removing EXIF metadata, including date and time of acquisition, camera settings, and GPS coordinates. Moreover, the same acquisition parameters, including manual/automatic mode, exposure time, aperture, ISO sensitivity, and focal length, were applied to ensure chromatic uniformity and stylistic consistency across all selected images, without altering lesion morphology or color patterns. No image editing, enhancement, cropping, filtering, or other post-processing was performed before the images were uploaded to the AI platform.

### 2.4. Clinical Data Collection

For all patients, standardized medical history and clinical data were collected, including age, sex, systemic comorbidities, current drug therapies, and potentially relevant deleterious habits, particularly tobacco smoking and alcohol use. In addition, for each lesion analyzed, the main clinical information was recorded, including time of onset, possible evolution over time, presence or absence of symptoms, and the clinical features observable at the time of examination, such as anatomical site, size, morphology, color, and surface appearance. Previous pathological samplings referring to the same lesion were not included among the information provided to the AI system, in order to avoid potential bias in the software’s diagnostic process and to ensure that the diagnostic hypothesis was formulated exclusively on the basis of the clinical and medical history data together with the photographic analysis.

### 2.5. AI-Based Diagnostic Assessment

Each image was individually uploaded to an AI software platform (GPT-5.2 Thinking) together with the corresponding clinical and medical history data. AI-based assessments were performed between March and April 2026.

Before uploading the images to the platform, guidance prompts were prepared containing the most recent classifications of the analyzed macro-categories and the corresponding diagnostic criteria according to the World Health Organization (WHO) [8,23,24] (Supplementary Table S2). For each case, the system was asked to provide the most likely diagnostic hypothesis on the basis of the clinical and medical history information provided, together with the photographic analysis. In order to enhance data protection and minimize data retention, the analyses performed with GPT-5.2 Thinking were carried out using the Temporary Chat function; according to the platform policy applicable during the study period, Temporary Chats were not used for model training and were automatically deleted from the platform’s systems within 30 days. Moreover, taking into account the intrinsic variability of GPT-5.2 Thinking responses to the same query, each request was repeated three times at three different times of the day, initiating a new GPT-5.2 Thinking session for each instance and preliminarily uploading the classificatory and diagnostic prompts. The final AI diagnosis was defined as the diagnosis most frequently provided across the three outputs. When at least two of the three independent AI outputs provided the same diagnosis, this diagnosis was considered the final AI-generated diagnosis. When all three outputs differed, no final diagnosis was assigned and the case was classified as having no diagnostic consensus (Image 1 in Supplementary Table S3). The responses obtained were recorded in Supplementary Table S3, including, for each patient, the clinical images submitted to the AI, the patient’s age and sex, the clinical information considered relevant for the diagnosis (according to the AI), the clinical and histological diagnosis, the correspondence between the histological diagnosis and the diagnosis provided by the AI, and, in case of non-correspondence, the alternative diagnostic hypothesis proposed by the AI.

### 2.6. Statistical Analysis

Statistical analysis was performed to assess the diagnostic performance of the AI-based test in identifying OPMDs within the overall sample of oral lesions. The final clinical and/or pathological diagnosis was used as the reference standard. No missing data were present for the index test or reference standard among the included cases.

AI-generated diagnoses were dichotomized as OPMDs or non-OPMDs and classified as true positives (TP), true negatives (TN), false positives (FP), or false negatives (FN). Sensitivity, specificity, overall accuracy, positive predictive value (PPV), and negative predictive value (NPV) were calculated to evaluate the ability of the AI system to distinguish OPMDs from other oral lesions. A secondary analysis was conducted to assess the diagnostic performance of the AI system for each specific OPMDs subtype. Each subtype-specific analysis was conducted across the overall sample of 103 lesions, using a one-versus-rest approach: the subtype of interest was considered positive, whereas all remaining diagnoses were considered negative. AI-generated diagnoses were dichotomized accordingly and classified as TP, TN, FP, or FN using the final clinical and/or pathological diagnosis as the reference standard. Subtype-specific sensitivity, specificity, overall accuracy, PPV, and NPV were calculated to evaluate the ability of the AI system to correctly identify each OPMD subtype. Finally, a further analysis was conducted to assess the diagnostic performance of the AI system in identifying OC within the overall sample of oral lesions. AI-generated diagnoses were dichotomized as OC or non-OC and classified as TP, TN, FP, or FN using the final clinical and/or pathological diagnosis as the reference standard. Sensitivity, specificity, overall accuracy, PPV, and NPV were calculated to evaluate the ability of the AI system to distinguish OC from other oral lesions. Agreement between the final AI-generated classification and the clinical and/or pathological reference standard was assessed using two separate unweighted Cohen’s kappa coefficients. The first analysis evaluated agreement for the binary classification of oral potentially malignant disorders versus non-OPMD lesions. The second evaluated agreement for the binary classification of oral carcinomas versus non-carcinoma lesions. Cohen’s kappa analyses were conducted separately and did not represent a simultaneous multi-categorical classification. Exact binomial 95% confidence intervals for sensitivity, specificity, accuracy, PPV, and NPV were calculated using the Clopper–Pearson method. Confidence intervals for Cohen’s kappa coefficients were calculated using the asymptotic standard error method. All statistical analyses were performed using R version 4.6.1 (R Foundation for Statistical Computing, Vienna, Austria).

## 3. Results

A total of 103 clinical images of oral lesions were included in the analysis. According to the reference standard, 69 lesions were classified as OPMDs, whereas 34 were classified as non-OPMDs lesions, including OC and other oral mucosal diseases. When diagnoses were dichotomized as OPMDs or non-OPMDs, the AI system correctly classified 64 of 69 OPMDs as OPMDs (TP) and classified 30 of 34 non-OPMDs lesions as non-OPMDs (TN). Five OPMDs cases were classified as non-OPMDs lesions (FN), whereas 4 non-OPMDs lesions were classified as OPMDs (FP). This resulted in a sensitivity of 92.75% (95% CI: 83.89–97.61%), a specificity of 88.24% (95% CI: 72.55–96.70%), and an overall binary classification accuracy of 91.26% (95% CI: 84.06–95.93%). The PPV was 94.12% (95% CI: 85.62–98.37%), whereas the NPV was 85.71% (95% CI: 69.74–95.19%). For the classification of OPMDs versus non-OPMD lesions, the observed agreement between the AI system and the reference standard was 91.26%. Cohen’s kappa was 0.804 (95% CI: 0.682–0.926), showing a high level of agreement beyond chance. In subtype-specific one-versus-rest analyses conducted across the overall sample, diagnostic performance varied among OPMD categories. HOL was correctly identified in 9 of 11 cases (TP), with 2 FN results, yielding a sensitivity of 81.82% (95% CI: 48.22–97.72%) and a specificity of 100% (95% CI: 96.07–100%). Diagnostic accuracy was 98.06% (95% CI: 93.16–99.76%). The PPV was 100% (95% CI: 66.37–100%), while the NPV was 97.87% (95% CI: 92.52–99.74%). NHOL was correctly diagnosed in 11 of 14 cases (TP), with 3 FN results. Sensitivity was 78.57% (95% CI: 49.20–95.34%), and specificity was 96.63 (95% CI: 90.46–99.30%). Diagnostic accuracy was 94.17% (95% CI: 87.75–97.83%). The PPV was 78.57% (95% CI: 49.20–95.34%), while the NPV was 96.63% (95% CI: 90.46–99.30%). OLP, the most represented OPMD category, was correctly identified in 19 of 22 cases (TP), with 3 FN, yielding a sensitivity of 86.36% (95% CI: 65.09–97.09%) and a specificity of 98.77% (95% CI: 93.31–99.97%). Diagnostic accuracy was 96.12% (95% CI: 90.35–98.93%). The PPV was 95% (95% CI: 75.13–99.87%), while the NPV was 96.39% (95% CI: 89.80–99.25%). All included cases of PVL (5/5), AC (1/1), OLL (8/8), GVHD (2/2), SLE-related oral lesions (4/4), and PKAS (2/2) were correctly classified. Given the very small number of observations in several of these categories, these findings should be regarded as descriptive case-level observations rather than robust estimates of subtype-specific diagnostic performance.

Regarding the OC group, 25 lesions were included. Of these, 21 were correctly classified as OC (TP), whereas four were incorrectly classified as non-OC lesions (FN). Among the four misclassified cases, two OSCCs were classified as NHOL and erythroplakia, respectively; one OVC was classified as NHOL; and one BCC was classified as NHOL. All four false negative cases were therefore classified as OPMDs. Among the 78 non-OC lesions, two were incorrectly classified as OC (FP), whereas 76 were correctly classified as non-OC (TN). Sensitivity was 84.00% (95% CI: 63.92–95.46%), specificity was 97.44% (95% CI: 91.04–99.69%), and overall accuracy was 94.17% (95% CI: 87.75–97.83%). The PPV was 91.30% (95% CI: 71.96–98.93%), whereas the NPV was 95.00% (95% CI: 87.69–98.62%). For the classification of OC versus non-OC lesions, Cohen’s kappa was 0.837 (95% CI: 0.711–0.963).

## 4. Discussion

GPT-5.2 Thinking showed promising diagnostic performance in distinguishing OPMDs from non-OPMD lesions in this retrospective, case-enriched dataset. The multimodal AI system correctly classified 64 of 69 OPMDs and 30 of 34 non-OPMDs lesions, supporting its potential future use as an adjunctive screening tool in oral medicine. Diagnostic performance, however, varied across the most frequently represented OPMDs subtypes. Sensitivity was 81.82% for HOL, 78.57% for NHOL, and 86.36% for OLP. The OLP cases that were most commonly missed were the atrophic-erosive forms, whereas good diagnostic performance was observed for the “white” variants. A similar trend emerged from the third statistical analysis, which focused on the OC group. The AI incorrectly classified four OC as OPMDs, three of which were identified as NHOL.

This finding suggests that the system may recognize the potentially malignant nature of certain lesions while underestimating the presence of an already established invasive neoplasm. This form of clinical downstaging therefore appears to occur primarily in lesions with heterogeneous visual patterns that may mimic, for example, NHOL or erythroplakic lesions. Complete diagnostic agreement was observed for PVL, AC, OLL, GVHD, oral lesions associated with SLE, and PKAS. This finding may, at least in part, be related to the relatively distinctive clinical and morphological features that can characterize some of these conditions. However, given the small number of cases included in several of these subgroups, this interpretation remains tentative and should be confirmed in larger datasets. Accordingly, apparently perfect classification in categories represented by one or very few cases should not be interpreted as evidence of reliable subtype-specific sensitivity or specificity, but only as descriptive findings within this exploratory dataset.

The findings of the present study are broadly consistent with previous evidence on AI-assisted analysis of clinical images of oral lesions [16,20,25]. In a systematic review and meta-analysis of 17 studies, Li et al. evaluated different machine-learning algorithms designed to provide diagnostic information on specific oral lesions of interest, including OPMDs and OC [13]. The review reported pooled sensitivity and specificity values of 89.9% and 89.2%, respectively. The pooled sensitivity was slightly lower than that observed in our study, whereas specificity was comparable [13]. A recent meta-analysis of six studies conducted by Horvath et al. focused specifically on the identification of OPMDs from intraoral photographs using several AI-based tools, including HRNet-W18, VGG19, InceptionV3, CranioCatch, EfficientNetB3, and ResNet-152 V2 [20]. The study reported a pooled mean sensitivity of approximately 93–94% and a specificity of 95% [20]. These estimates are comparable to the sensitivity observed in the present investigation, although the specificity calculated in our study was moderately lower. However, in the meta-analysis by Horvath et al., the results with the highest sensitivity were selected from the best-performing AI models [20]. The six included studies used either a single AI model or, in some cases, two, three, or four different models to analyse the images [20]. Furthermore, all six studies employed dedicated image-analysis software, some of which had been specifically developed for dental imaging, such as CranioCatch [20]. Finally, Horvath et al. considered OPMDs exclusively. By contrast, our study also included OC and a heterogeneous group of inflammatory, autoimmune, infectious, and reactive lesions. Consequently, our dataset may have presented the AI with a more complex diagnostic task, particularly for heterogeneous lesions and those with borderline features. Direct comparisons between predictive values should therefore be interpreted with caution. Horvath et al. reported a PPV of 42% and an NPV of 99%. In our cohort, OPMDs accounted for 69 of 103 cases, resulting in a substantially higher PPV of 94.12%, but a lower NPV of 85.71% [20]. Because PPV and NPV are strongly influenced by disease prevalence, these estimates should be interpreted within the context of the deliberately case-enriched composition of our dataset. In particular, the high proportion of OPMDs is likely to have increased PPV while reducing NPV relative to what might be observed in an unselected primary-care or population-screening setting. Therefore, these predictive values should not be extrapolated to routine clinical populations and require prospective validation in cohorts reflecting real-world disease prevalence.

The diagnostic performance observed in our study was superior to that reported in earlier investigations assessing general-purpose Large Language Models (LLMs) rather than purpose-built Convolutional Neural Networks (CNNs). Pradhan et al. reported that Chat-GPT-4 correctly identified 28 of 42 cases (66.6%) of OPMDs and OC when clinical photographs were combined with clinical descriptions. In that study, dentists with expertise in AI achieved better diagnostic performance when providing their own clinical diagnoses [26]. Similarly, Suárez et al. reported low sensitivity and diagnostic accuracy for Chat-GPT-4o in an analysis of 23 lesions suspected to represent OSCC, HOL, or OLP. Nevertheless, the use of Chat-GPT-4o already showed promising potential as a complementary tool for clinical OSCC screening based on imaging [19]. By comparison, the high classification performance achieved in our study may be attributable to the use of standardised clinical photographs; a more recent software version, namely Chat-GPT-5.2, released in December 2025; detailed patient-specific clinical information; targeted guiding prompts; and a majority-voting procedure conducted across three separate sessions. The benefit of combining clinical photographs with clinical information is supported by the findings of Schmidl et al. In their assessment of Chat-GPT-4.0, the addition of clinical history increased sensitivity for OSCC from 18.2% to 100% and specificity from 52.2% to 88.2%. For OL, sensitivity increased from 72.2% to 93.3%, while specificity increased from 92.6% to 96.7% [27]. However, the study did not provide several relevant methodological details, including whether previous pathological diagnoses or clinical data that were already highly indicative of the final diagnosis had been included. The availability of such information may have contributed to the remarkably high sensitivity values reported. A recent study by Vaira et al., published in 2025 and based on 110 images obtained from the internet, found that Chat-GPT-4.0 provided the exact diagnosis in 85% of cases and distinguished malignant from benign lesions with a sensitivity of 91.7% and a specificity of 100% [28]. However, unlike the study by Vaira et al., which used a binary benign-versus-malignant endpoint, our dataset included several borderline lesions located along the clinical continuum between OPMDs and OC, and the AI was specifically required to identify and distinguish these diagnostic categories.

The accuracy of 91.26% suggests that GPT-5.2 Thinking may have potential as a diagnostic support for frontline healthcare professionals, although this potential requires prospective evaluation against human comparators [14]. If prospectively validated, the model could act as an automated warning system, supporting less experienced clinicians in the early detection of suspicious lesions, reducing diagnostic uncertainty, and potentially limiting delays in diagnosis [29].

When integrated into telemedicine and digital health platforms, such an algorithm could enable rapid, cost-effective, and standardised screening, including in remote geographical areas or settings lacking healthcare facilities equipped to perform an appropriate oral examination.

Future integration into telemedicine and digital health platforms could represent a potential application of this approach; however, its effectiveness for remote or population-based screening remains to be established in prospective studies.

This approach could facilitate and accelerate the referral of at-risk patients to specialised oral medicine centres [14].

The present study is distinguished within the current literature by several methodological strengths. First, the inclusion of a heterogeneous range of oral diseases allowed the AI system to be evaluated across a broad spectrum of lesions encountered in specialist oral medicine and oral pathology practice, although the case-enriched composition of the sample does not reflect their prevalence in routine clinical settings.

Furthermore, the high sensitivity observed for OPMD identification suggests that the model may have potential as an adjunctive tool for recognising lesions requiring further specialist assessment. However, these findings should be regarded as preliminary and require confirmation in prospective, unselected, and externally validated clinical cohorts. Nevertheless, the study has several limitations, including its single-centre design and the evaluation of a single AI system. Furthermore, the exclusion of poor-quality photographs may have resulted in an optimized image dataset and could therefore have overestimated diagnostic performance compared with routine clinical or telemedicine settings.

Because image and clinical data were provided together, the present study cannot disentangle their individual contributions to diagnostic performance.

In addition, despite the overall heterogeneity of the included conditions, some disease-specific subgroups were small, such as AC, represented by one case, GVHD by two cases, and SLE by four cases. These limited sample sizes warrant caution when extrapolating and generalising the corresponding performance estimates. The algorithm is inherently unable to assess the three-dimensional features of a lesion or obtain tactile information. In particular, it cannot perform clinical palpation, a fundamental component of physical examination that allows clinicians to evaluate tissue texture and consistency, which may represent a key diagnostic feature of certain malignant neoplasms of the oral cavity. Moreover, the AI algorithm correctly identified 21 of the 25 neoplastic lesions included in the study, but nevertheless produced four FN results. This finding highlights a critical limitation and indicates that AI systems have not yet achieved absolute sensitivity in the detection of oral neoplastic diseases. It also confirms the persistent difficulty in correctly identifying borderline lesions with heterogeneous textures. Computational technology should therefore not be regarded as a replacement for objective clinical examination, but rather as a complementary screening tool.

## 5. Conclusions

In conclusion, the introduction of AI into oral medicine and oral pathology represents a paradigm shift that places the synergistic interaction between clinicians and technology at the centre of patient care. AI should not be regarded as a replacement for clinical judgement, but rather as a supportive tool aimed at improving early diagnosis and patient management [22,30]. For this synergy to be effectively implemented in routine clinical practice, future research must move beyond the technical validation of software under controlled conditions [22]. Instead, it will be essential to assess the real-world impact of AI on clinician performance through standardised comparative studies capable of quantifying the added value of this technology over conventional clinical practice [20,22].

Furthermore, the predictive values reported in the present study should be considered specific to this case-enriched dataset and should not be extrapolated to routine clinical populations without prospective validation in cohorts with representative disease prevalence. In this context, human factors, including clinical experience and the learning curve of professionals using AI-based software, are likely to play a decisive role in diagnostic performance [30]. Over the coming years, it will therefore be crucial to establish an international methodological consensus that standardises these processes and ensures that AI is integrated into clinical practice in a safe, effective, and scientifically grounded manner.

## Data Availability

The original contributions presented in this study are included in the article/Supplementary Materials. Further inquiries can be directed to the corresponding author.

## Supplementary Materials

The following supporting information can be downloaded at: https://www.mdpi.com/article/10.3390/dj14100657/s1, Table S1: STARD-AI item; Table S2: Current Classification and Diagnostic References for Photographable Oral Conditions; Table S3: Patients clinical data.
