# Supplementary material for: Artificial Intelligence-Based Evaluation of Oral Potentially Malignant Disorders Using Photographic and Clinical Data: A Retrospective Exploratory Diagnostic Accuracy Study
Source: Dent J (Basel). 2026 Oct 10;14(10):657. doi: 10.3390/dj14100657 (PMC13703694; doi:10.3390/dj14100657)
Supplement: Supplementary file 1 [file dentistry-14-00657-s001.zip › dentistry-4551901-supplementary.pdf]

Supplementary Table S1: Downloadable version of the STARD-AI checklist

Section and Topic	No.	STARD-AI Item	Reported on page
**Title or abstract**			
	1	Identification as a study reporting AI-centred diagnostic accuracy and reporting at least one measure of accuracy within title or abstract	1
**Abstract**			
	2	Structured summary of study design, methods, results and conclusions (for specific guidance, please see STARD for Abstracts)	1
**Introduction**			
	3	Scientific and clinical background, including the intended use of the index test, whether it is novel or an established index test, and its integration into an existing or new workflow, if applicable	1-2
	4	Study objectives and hypotheses	2-3
**Methods**			
Study design	5	Whether data collection was planned before the index test and reference standard were performed (prospective study) or after (retrospective study)	3
Ethics	6	Formal approval from an ethics committee. If not required, justify why	3; 11
Participants	7	Eligibility criteria: listing separate inclusion and exclusion criteria in the order that they are applied at both participant level and data level	3-4
	8	On what basis potentially eligible participants were identified (such as symptoms, results from previous tests, inclusion in registry)	3-4
	9	Where and when potentially eligible participants were identified (setting, location, and dates)	3
	10	Whether participants formed a consecutive, random, or convenience series	4
Dataset	11	Source of the data and whether it has been routinely collected, specifically collected for the purpose of the study or acquired from an open-source repository	3-4
	12	Who undertook the annotations for the dataset (including experience levels and background) and how (within the same clinical context or in a post-hoc fashion), if applicable	5
	13	Devices (manufacturer, model) that were used to capture data; software (with version number) used to engineer the index test, highlighting the intended use	3; 5
	14	Data acquisition protocols (e.g. contrast protocol or reconstruction method for medical images) and details of data pre-processing in sufficient detail to allow replication	5
Test methods	15a	Index test, in sufficient detail to allow replication	5-6
	15b	How the index test was developed, including any training, validation, testing and external evaluation, detailing sample sizes, when applicable	N/A
	15c	Definition of and rationale for test positivity cut-offs or result categories of the index test, distinguishing pre-specified from exploratory	6
	15d	The specified end user of the index test and the level of expertise required of users	9-10
	16a	Reference standard, in sufficient detail to allow replication	5

Supplementary Table S2.

Current Classification and Diagnostic References for Photographable Oral Conditions

Photographable conditions covered	Current classification or diagnostic reference	DOI or official source
**Universal coding umbrella — all diseases and disorders of the oral cavity, lips, salivary glands, teeth, periodontium and jaws**	WHO ICD-11 for Mortality and Morbidity Statistics (current online release). Useful as an overarching coding system; it is not a photo-based diagnostic standard.	No DOI — WHO ICD-11
**Oral potentially malignant disorders (OPMDs) — leukoplakia, erythroplakia, proliferative verrucous leukoplakia, oral submucous fibrosis, actinic cheilitis, palatal lesions in reverse smokers, dyskeratosis congenita, oral lichen planus and selected lichenoid disorders**	WHO Collaborating Centre consensus report on nomenclature and classification of OPMDs (2021).	10.1111/odi.13704
**Oral epithelial dysplasia and malignant epithelial tumours — dysplasia, carcinoma in situ, conventional oral squamous cell carcinoma, verrucous carcinoma and related variants**	WHO Classification of Head and Neck Tumours, 5th ed., Vol. 9 (2024), plus the WHO oral cavity/mobile tongue update.	No DOI — official IARC/WHO volume<br>10.1007/s12105-021-01402-9
**Oral and lip melanocytic, soft-tissue, neural, haematolymphoid and metastatic tumours — including oral melanoma, Kaposi sarcoma, lymphoma and visible metastases**	WHO Classification of Head and Neck Tumours, 5th ed., Vol. 9 (2024).	No DOI — official IARC/WHO volume
**Lip and perioral cutaneous tumours — basal cell carcinoma, cutaneous squamous cell carcinoma, melanocytic and adnexal tumours**	WHO Classification of Skin Tumours, 5th ed., Vol. 12 (2025).	No DOI — official IARC/WHO volume
**Major and minor salivary-gland tumours — benign and malignant epithelial, myoepithelial and other salivary neoplasms**	WHO Classification of Head and Neck Tumours, 5th ed. (2024), with the fifth-edition salivary-gland update.	No DOI — official IARC/WHO volume<br>10.1007/s12105-022-01420-1
**Odontogenic and maxillofacial bone tumours — visible gingival/alveolar presentations of odontogenic tumours, cystic lesions and jaw tumours**	WHO Classification of Head and Neck Tumours, 5th ed. (2024), with the fifth-edition odontogenic/maxillofacial bone update.	No DOI — official IARC/WHO volume<br>10.1007/s12105-021-01404-7

**Oral lichen planus and oral lichenoid lesions — reticular, plaque-like, atrophic, erosive, bullous and mixed patterns**	American Academy of Oral and Maxillofacial Pathology position paper and diagnostic criteria (2016).	10.1016/j.oooo.2016.05.004
**Recurrent aphthous stomatitis and complex aphthosis — minor, major and herpetiform ulcers**	No universally accepted stand-alone international diagnostic criteria. Use clinicopathologic assessment and exclude systemic causes; code within ICD-11 where appropriate.	No stand-alone DOI — WHO ICD-11
**Traumatic, frictional, factitial, chemical, thermal and contact lesions — traumatic ulcer, frictional keratosis, morsicatio, linea alba, contact stomatitis and burns**	No single international classification or validated photo-only diagnostic criteria. Use descriptive clinicopathologic diagnosis and ICD-11 coding.	No stand-alone DOI — WHO ICD-11
**Benign reactive and exophytic lesions — irritation fibroma, giant-cell fibroma, pyogenic granuloma, peripheral giant-cell granuloma, peripheral ossifying fibroma, epulis and verruciform xanthoma**	Covered by current clinicopathologic terminology and, for neoplastic entities, WHO Head and Neck Tumours. No universal photo-only criteria.	No stand-alone DOI<br>Official IARC/WHO volume
**Normal variants and developmental mucosal/tongue findings — Fordyce granules, leukoedema, fissured tongue, geographic tongue, coated/hairy tongue, lingual varices, tori and exostoses**	No single international disease classification or validated photo-only criteria. Use standard clinical terminology and ICD-11 when a code exists.	No stand-alone DOI — WHO ICD-11
**Oral candidiasis — pseudomembranous, erythematous, chronic hyperplastic, angular cheilitis and Candida-associated lesions**	Global guideline for the diagnosis and management of candidiasis (ECMM/ISHAM/ASM; 2025). Oral subtype diagnosis remains clinicomicrobiological.	10.1016/S1473-3099(24)00749-7
**Viral oral lesions — herpes simplex, varicella-zoster, HPV-related papilloma/verruca/condyloma/focal epithelial hyperplasia, EBV oral hairy leukoplakia, CMV, enteroviral disease and mpox**	No single oral-specific international classification. Use ICD-11 and pathogen-specific diagnostic standards; morphology alone is not definitive.	No single DOI — WHO ICD-11
**Bacterial infections — syphilis, tuberculosis, actinomycosis, gonococcal disease, noma and other bacterial stomatitides**	Use disease-specific standards and ICD-11. The 2020 European guideline is the current cited reference for syphilis.	10.1111/jdv.16946<br>WHO ICD-11
**Deep fungal and parasitic oral lesions — histoplasmosis, blastomycosis, cryptococcosis, mucormycosis, leishmaniasis and related infections**	No single oral-specific classification. Use ICD-11 and organism-specific microbiological/histopathological standards.	No single DOI — WHO ICD-11

**Mucous membrane pemphigoid**	European S3 guideline, Part I: definition, outcome measures and clinical/laboratory diagnosis (2021).	10.1111/jdv.17397
**Pemphigus vulgaris and pemphigus foliaceus**	International panel recommendations for diagnosis and management (2020 publication).	10.1016/j.jaad.2018.02.021
**Paraneoplastic pemphigus / paraneoplastic autoimmune multiorgan syndrome**	Revised diagnostic criteria based on literature analysis and expert evaluation (2021).	10.1111/cup.14004
**Other autoimmune blistering diseases — bullous pemphigoid, epidermolysis bullosa acquisita, linear IgA disease and dermatitis herpetiformis**	No unified oral-photo classification. Diagnosis requires disease-specific clinical, histological and direct/indirect immunofluorescence criteria.	No single DOI
**Erythema multiforme and severe drug-related mucocutaneous reactions — Stevens–Johnson syndrome and toxic epidermal necrolysis**	Use clinicopathologic/drug-causality assessment. Current German S3 guideline for SJS/TEN, Part I (2024).	10.1111/ddg.15515
**Oral chronic graft-versus-host disease — lichenoid, erythematous, ulcerative, mucoceles and salivary dysfunction phenotypes**	NIH Consensus Development Project: 2014 diagnosis and staging report (published 2015).	10.1016/j.bbmt.2014.12.001
**Systemic lupus erythematosus and lupus-related oral lesions**	2019 EULAR/ACR classification criteria for SLE. These are classification—not stand-alone diagnostic—criteria.	10.1002/art.40930
**Behçet disease — recurrent oral/genital ulcers and mucocutaneous findings**	International Criteria for Behçet's Disease (ICBD; 2014 publication).	10.1111/jdv.12107
**Sjögren disease — xerostomia-associated oral findings and salivary-gland disease**	2016 ACR/EULAR classification criteria (published 2017). These are classification criteria.	10.1002/art.39859
**IgG4-related disease — salivary-gland enlargement and oral manifestations**	2019 ACR/EULAR classification criteria. These are classification criteria.	10.1136/annrheumdis-2019-216561
**Sarcoidosis — nodular, papular, gingival and salivary presentations**	American Thoracic Society clinical practice guideline for diagnosis and detection (2020).	10.1164/rccm.202002-0251ST
**Amyloidosis — macroglossia, nodules, plaques, petechiae and other oral deposits**	Amyloid fibril protein nomenclature recommendations (2024). This classifies amyloid proteins; diagnosis still requires tissue confirmation.	10.1080/13506129.2024.2405948
**Gastrointestinal, haematologic, renal, endocrine and nutritional disease with oral signs — Crohn disease/orofacial granulomatosis, coeliac disease,**	No single oral-specific international classification. Use the primary disease criteria plus ICD-11; oral	No single DOI — WHO ICD-11

**anaemia, leukaemia, neutropenia, thrombocytopenia, uraemic stomatitis, Addison disease and deficiencies**	photographs alone cannot establish the systemic diagnosis.	
**Cancer therapy–related oral mucositis**	MASCC/ISOO clinical practice guidelines for mucositis secondary to cancer therapy (2020).	10.1002/cncr.33100
**Other medication-related oral lesions — lichenoid reactions, ulcers, pigmentation, gingival enlargement, salivary change and immunotherapy-associated lesions**	No unified international classification. Diagnose by phenotype, temporal association, exclusion and drug-specific criteria; code in ICD-11 where applicable.	No single DOI — WHO ICD-11
**Medication-related osteonecrosis of the jaw (MRONJ) — exposed bone or bone probed through a fistula, including visible intraoral stages**	American Association of Oral and Maxillofacial Surgeons position paper, 2022 update.	10.1016/j.joms.2022.02.008
**Osteoradionecrosis and other exposed-jaw-bone disorders**	No single universally adopted classification for all causes. Use cause-specific clinical/imaging criteria and ICD-11; photography documents only the visible component.	No single DOI — WHO ICD-11
**Pigmented oral lesions — physiologic pigmentation, melanotic macule, smoker/drug/post-inflammatory pigmentation, amalgam tattoo, naevi, melanoma, Kaposi sarcoma and systemic pigmentation**	WHO Head and Neck Tumours and WHO Skin Tumours cover neoplastic entities; benign and systemic pigmentation has no single photo-only classification.	No DOI — WHO Head and Neck Tumours<br>No DOI — WHO Skin Tumours
**Vascular anomalies — haemangiomas, venous/lymphatic/capillary/arteriovenous malformations and syndromic vascular lesions**	ISSVA Classification of Vascular Anomalies, updated 2025.	10.1097/JOVA.000000000000011 3<br>Official ISSVA classification
**Non-neoplastic salivary and ductal lesions — mucocele, ranula, sialolith at a duct opening, sialadenitis, necrotising sialometaplasia and salivary hypofunction**	No single international photo-based classification. Use clinicopathologic/imaging criteria, disease-specific standards and ICD-11.	No single DOI — WHO ICD-11
**Oral burning and pain disorders with possible visible correlates — burning mouth disorder, persistent idiopathic facial pain, painful cranial neuropathies and mucosal pain**	International Classification of Orofacial Pain, first edition (ICOP; 2020).	10.1177/0333102419893823
**Temporomandibular disorders with photographable extraoral/intraoral findings**	Diagnostic Criteria for Temporomandibular Disorders (DC/TMD; 2014).	10.11607/jop.1151

**Gingival, periodontal and peri-implant diseases — plaque-induced and non-plaque-induced gingival disease, periodontitis, necrotising periodontal disease, peri-implant mucositis and peri-implantitis**	2017 World Workshop classification of periodontal and peri-implant diseases and conditions (published 2018).	10.1111/jcpe.12935
**Dental caries — non-cavitated and cavitated coronal/root lesions visible clinically or photographically**	International Caries Detection and Assessment System (ICDAS).	10.1111/j.1600-0528.2007.00347.x
**Erosive tooth wear and other non-carious tooth wear — erosion, attrition and abrasion**	Basic Erosive Wear Examination (BEWE; 2008). BEWE is mainly a screening system for erosive tooth wear.	10.1007/s00784-007-0181-5
**Developmental defects of enamel and dentine — molar-incisor hypomineralisation, fluorosis, enamel hypoplasia, amelogenesis imperfecta and dentinogenesis imperfecta**	Updated EAPD policy for MIH (2022); other inherited/developmental defects require condition-specific clinical/genetic classification and ICD-11.	10.1007/s40368-021-00668-5<br>WHO ICD-11
**Traumatic dental injuries — enamel/dentine fractures, crown-root/root fractures, luxation injuries and avulsion**	International Association of Dental Traumatology guidelines, Part 1: fractures and luxations (2020).	10.1111/edt.12578
**Congenital and craniofacial conditions visible in oral photographs — cleft lip/palate, ankyloglossia, macroglossia, microglossia and related anomalies**	Use ICD-11 and syndrome-specific diagnostic standards. No single oral-photo classification covers this entire group.	No single DOI — WHO ICD-11

Interpretive notes. "No DOI" may indicate an official WHO/IARC book or online classification that is authoritative but has no DOI. "No stand-alone DOI" means that no single internationally accepted classification or validated photo-only diagnostic standard was identified for the whole group. Rheumatology classification criteria are not equivalent to individual-patient diagnostic criteria.

Supplementary Table S3.

Patient clinical image	Sex/Age	Relevant medical history for diagnosis	Clinical Diagnosis	Histological Diagnosis	Diagnostic Agreement with AI	AI Diagnosis
**ORAL LEUKOPLAKIA**						
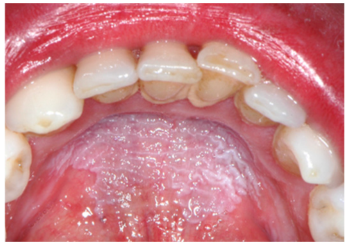	F/58	Smoker<br>(30 cpd)<br>Drinkers of 2 U/die<br>Floor of the mouth	HOL	Chronic inflammation (lichenoid), orthoparakeratosis	YES	
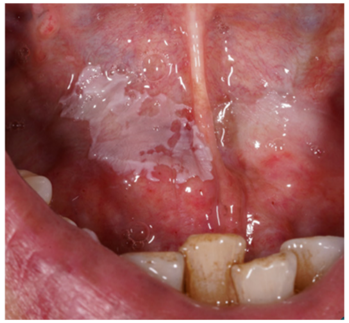	F/46	Smoker<br>(20 cpd)<br>Ventral tongue and floor of the mouth	HOL	Chronic inflammation, orthoparakeratosis	YES	
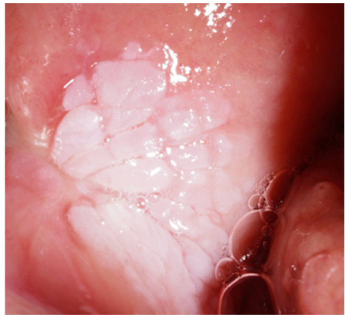	M/70	Drinker of 2 U/die<br>5 years Follow-up<br>Buccal Mucosa	HOL	Mild OED	YES	

Patient clinical image	Sex/Age	Relevant medical history for diagnosis	Clinical Diagnosis	Histological Diagnosis	Diagnostic Agreement with AI	AI Diagnosis
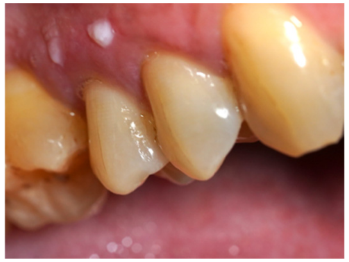	M/65		HOL	Hyperkeratosis	YES	
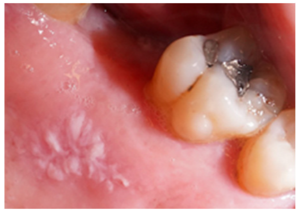	M/59	Ex smoker (20 cpd)<br>Hard Palate	HOL	Chronic inflammation, orthoparakeratosis	NO	OSP
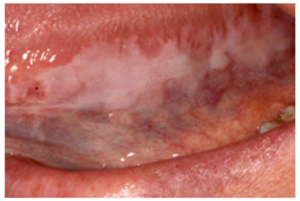	F/60	Bruxism<br>Smoker<br>(10 cpd)<br>Lateral tongue<br>3 years Follow-up	HOL	Mild OED	YES	
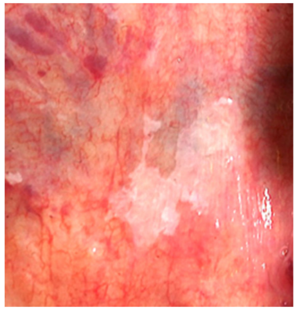	F/59	Smoker<br>(10 cpd)<br>Drinker of 1U/die<br>Lateral tongue	HOL	Mild OED	YES	

Patient clinical image	Sex/Age	Relevant medical history for diagnosis	Clinical Diagnosis	Histological Diagnosis	Diagnostic Agreement with AI	AI Diagnosis
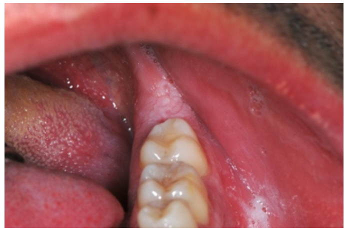	M/46		BARK/HOL	Hyperkeratosis, inflammatory infiltrate	YES	
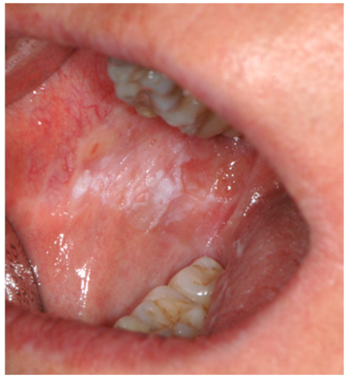	M/69	2 years Follow-up	HOL	Hyperkeratosis, acanthosis	YES	
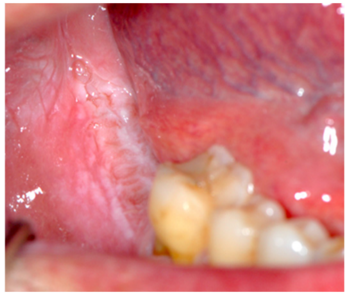	M/77		BARK/HOL	Hyperkeratosis, Inflammatory infiltrate	YES	

Patient clinical image	Sex/Age	Relevant medical history for diagnosis	Clinical Diagnosis	Histological Diagnosis	Diagnostic Agreement with AI	AI Diagnosis
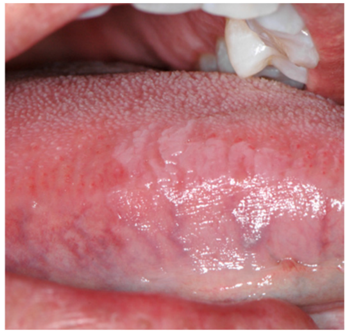	F/48	Pain<br>Lateral Tongue<br>4 years Follow-up	HOL	Hyperkeratosis	NO	FK
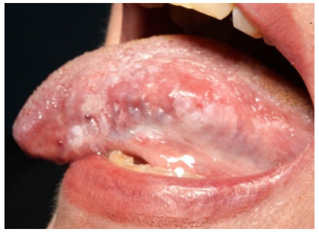	F/65	Pain<br>Burning<br>7 years Follow-up<br>Non-Homogeneous Lesion	NHOL	Moderate OED	YES	
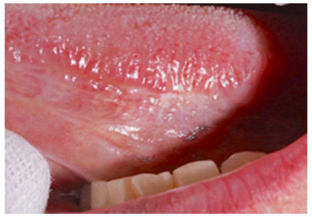	F/59	Lateral Tongue	NHOL	Mild OED	YES	
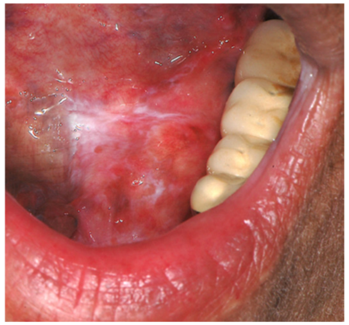	F/68	Floor of the mouth<br>Non-homogeneous Lesion<br>Smoker (15 cpd)	NHOL	Moderate OED	YES	

Patient clinical image	Sex/Age	Relevant medical history for diagnosis	Clinical Diagnosis	Histological Diagnosis	Diagnostic Agreement with AI	AI Diagnosis
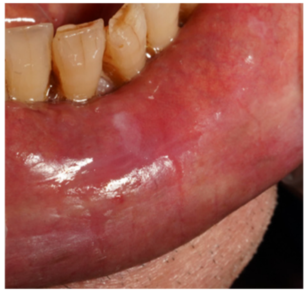	M/70		NHOL	Mild OED	YES	
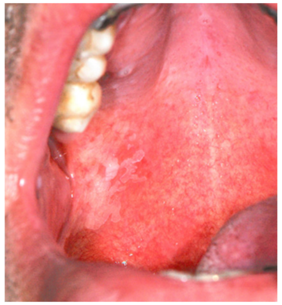	M/67		NHOL	Moderate OED	YES	
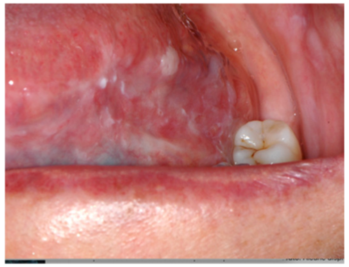	F/76	Smoker (20 cpd)<br>Pain<br>11 years Follow-up<br>Lateral Tongue<br>Non-homogeneous Lesion	Recurrent NHOL	Severe OED/CIS	YES	

Patient clinical image	Sex/Age	Relevant medical history for diagnosis	Clinical Diagnosis	Histological Diagnosis	Diagnostic Agreement with AI	AI Diagnosis
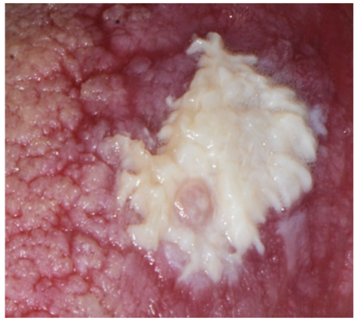	M/60	Ex smoker (40 cpd)<br>Drinker of 1U/die<br>Dorsal Tongue	NHOL	Severe OED	YES	
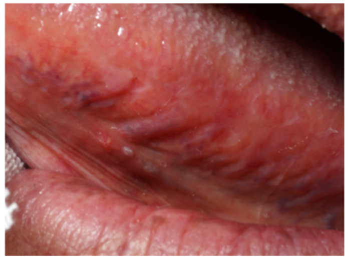	M/70	Drinker of 2 U/die<br>Lateral Togue<br>Non-homogeneous Lesion	NHOL	Moderate OED	YES	
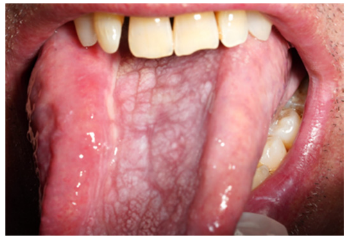	M/65	Burning<br>Pain	NHOL	Mild OED	NO	LPO

Patient clinical image	Sex/Age	Relevant medical history for diagnosis	Clinical Diagnosis	Histological Diagnosis	Diagnostic Agreement with AI	AI Diagnosis
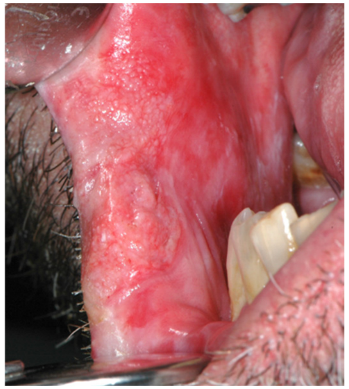	M/47	Burning<br>Pain<br>Non-homogeneous Lesion<br>6 years Follow-up	NHOL	Moderate OED	NO	OSCC
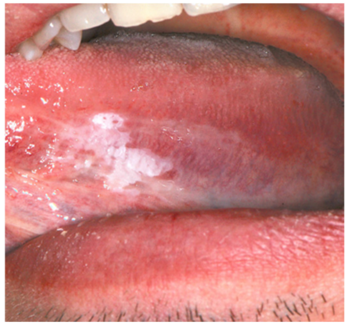	M/36	Drinker of 2 U/die<br>Lateral tongue	NHOL	Moderate OED	YES	
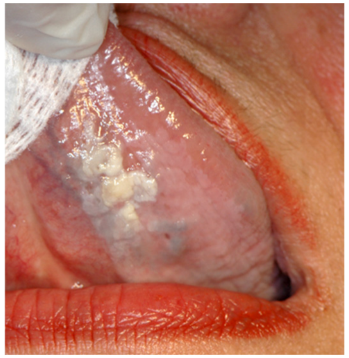	F/81	Burning<br>Ventral tongue	NHOL	Mild OED	YES	

Patient clinical image	Sex/Age	Relevant medical history for diagnosis	Clinical Diagnosis	Histological Diagnosis	Diagnostic Agreement with AI	AI Diagnosis
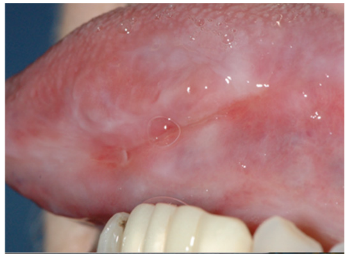	F/84	Burning<br>Non-homogeneous Lesion<br>12 years Follow-up	NHOL	Moderate OED	NO	OSCC
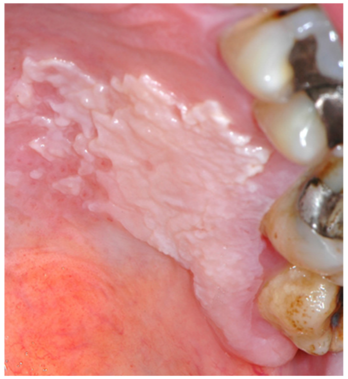	M/56	Smoker (10 cpd)<br>Mono-lateral Lesion	NHOL	Mild OED	YES	
**ORAL LICHEN PLANUS**						
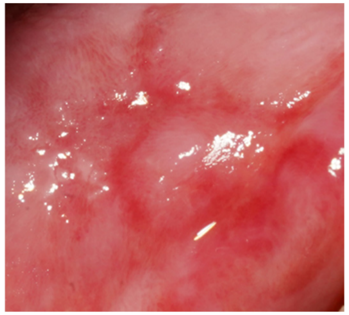	F/60	Burning<br>Pain<br>Bilateral Lesions<br>Buccal mucosa	OLP	OLP	YES	

Patient clinical image	Sex/Age	Relevant medical history for diagnosis	Clinical Diagnosis	Histological Diagnosis	Diagnostic Agreement with AI	AI Diagnosis
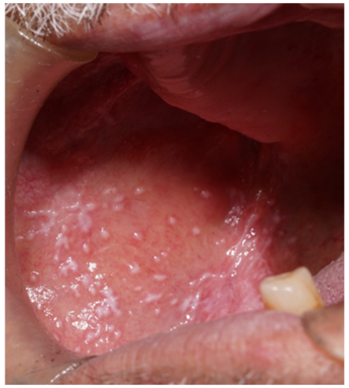	M/63	Ex smoker<br>(20 cpd)<br>Burning<br>Bilateral Lesions	OLP	OLP	YES	
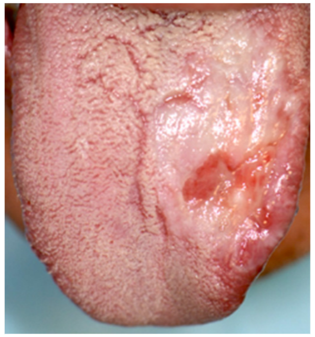	F/68	10 years Follow-up<br>Pain<br>Burning	OLP	OLP	YES	
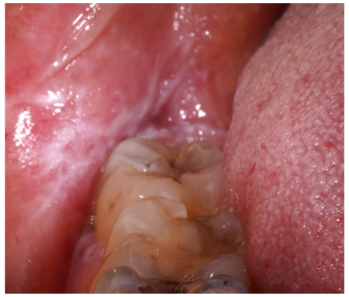	F/75	Smoker<br>(10 cpd)<br>Burning<br>Bilateral Lesions<br>5 years Follow-up	OLP	OLP	YES	

Patient clinical image	Sex/Age	Relevant medical history for diagnosis	Clinical Diagnosis	Histological Diagnosis	Diagnostic Agreement with AI	AI Diagnosis
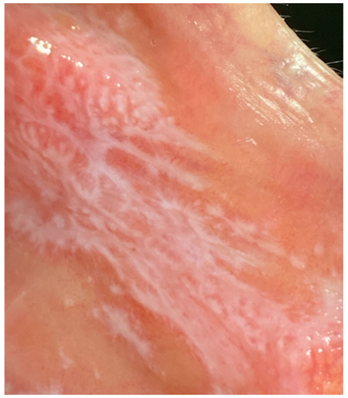	F/72	Burning<br>Bilateral Lesions	OLP	OLP	YES	
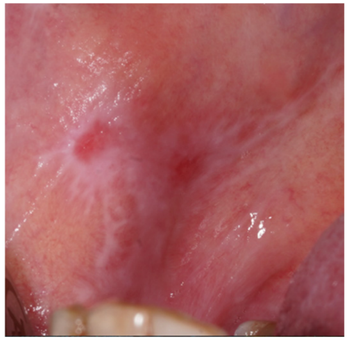	F/56	Pain<br>Bilateral Lesions<br>8 years Follow-up	OLP	OLP	YES	
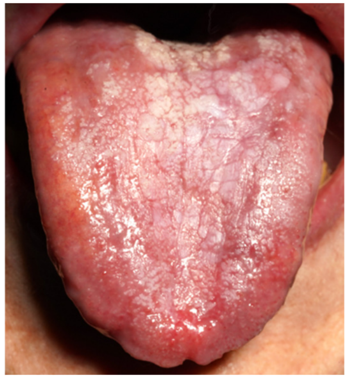	F/71	11 years Follow-up	OLP	OLP	YES	

Patient clinical image	Sex/Age	Relevant medical history for diagnosis	Clinical Diagnosis	Histological Diagnosis	Diagnostic Agreement with AI	AI Diagnosis
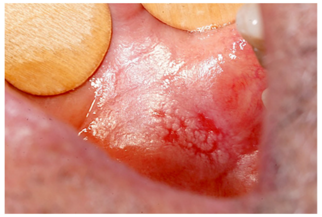	M/70	Ex smoker<br>Drinker of 1U/die<br>Bilateral Lesions on buccal mucosa<br>Pain<br>Burning	OLP	OLP	YES	
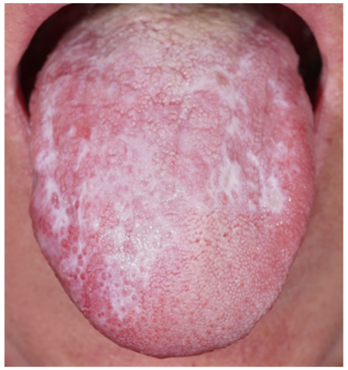	F/66	Burning<br>5-year Follow-up	OLP	OLP	YES	
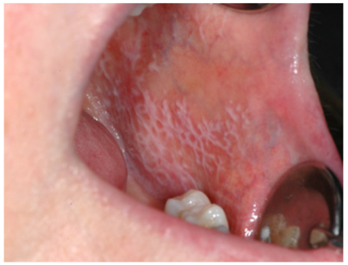	M/70	Burning<br>Pain<br>Bilateral Lesions	OLP	OLP	YES	
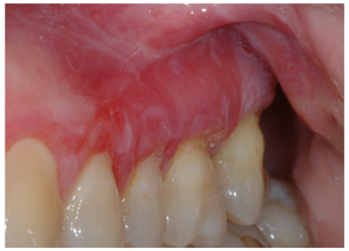	F/64	Burning	OLP	OLP	NO	EP

Patient clinical image	Sex/Age	Relevant medical history for diagnosis	Clinical Diagnosis	Histological Diagnosis	Diagnostic Agreement with AI	AI Diagnosis
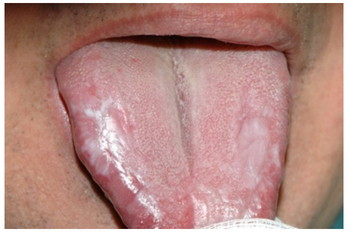	M/68	12 years Follow-up	OLP	OLP	YES	
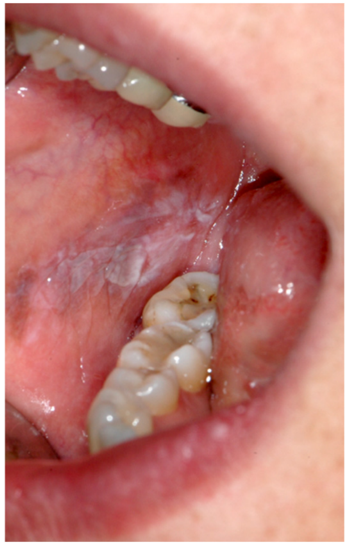	F/59	Bilateral Lesions<br>4 years Follow-up	OLP	OLP	YES	
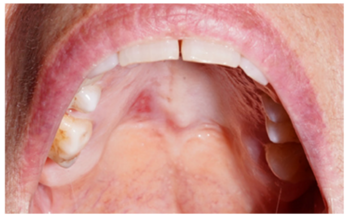	F/70	Burning<br>Pain	OLP	OLP	NO	ULCER

Patient clinical image	Sex/Age	Relevant medical history for diagnosis	Clinical Diagnosis	Histological Diagnosis	Diagnostic Agreement with AI	AI Diagnosis
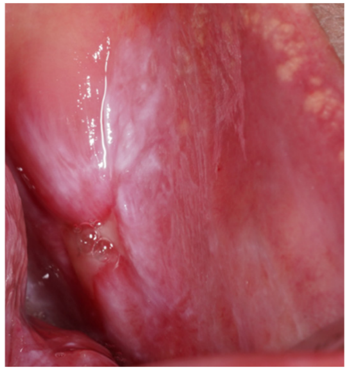	F/64	Burning<br>Pain<br>Bilateral Lesions on buccal mucosa	OLP	OLP	YES	
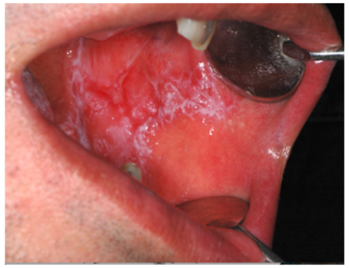	M/60	Bilateral buccal lesions<br>Pain<br>Burning<br>6 years Follow-up	OLP	OLP	YES	
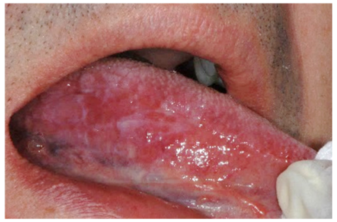	M/56	Drinker of 1 U/die<br>Bilateral Lesions<br>Burning<br>2 years Follow-Up	OLP	OLP	YES	
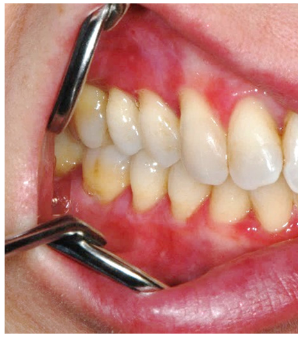	F/45	Burning<br>Pain<br>Bilateral Lesions	OLP	OLP	YES	

Patient clinical image	Sex/Age	Relevant medical history for diagnosis	Clinical Diagnosis	Histological Diagnosis	Diagnostic Agreement with AI	AI Diagnosis
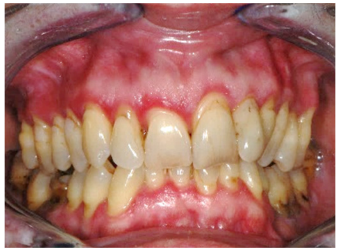	F/61	Smoker (20 cpd)<br>Burning<br>Pain<br>Xerostomia<br>Anaesthesia<br>Dysgeusia<br>Dysphagia	Desquamative gengivitis	OLP	YES	
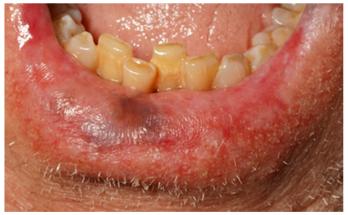	M/70	Smoker (10 cpd)<br>Burning	OLP	OLP	NO	AC
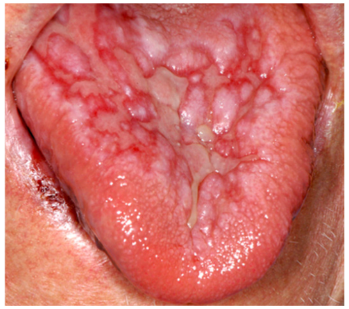	M/91	Burning<br>Pain<br>HCV+	OLP	OLP	YES	
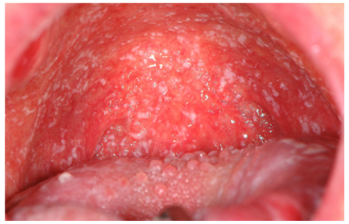	F/81	Burning<br>Xerostomia<br>Ex HCV+	OLP	OLP	YES	

Patient clinical image	Sex/Age	Relevant medical history for diagnosis	Clinical Diagnosis	Histological Diagnosis	Diagnostic Agreement with AI	AI Diagnosis
**PROLIFERATIVE VERRUCOUS LEUCOPLAKIA**						
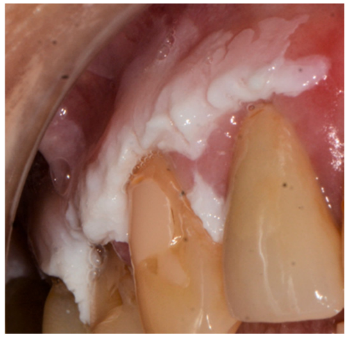	F/76	Drinker 2 U/die<br>Smokers (20 cpd)<br>Bilateral Lesions<br>8 years Follow-up	PVL	PVL	YES	
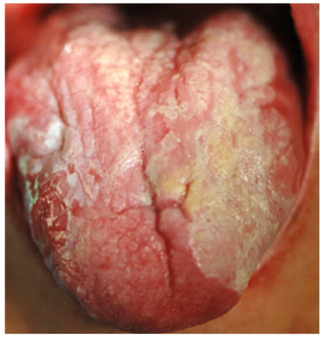	F/70	Burning	PVL	PVL	YES	
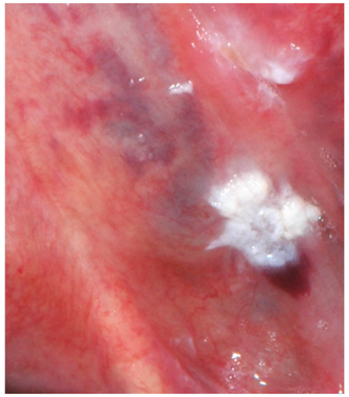	M/57	Smoker (15 cpd)<br>Ventral tongue<br>Bilateral Lesions<br>2 years Follow-up	PVL	PVL	YES	

Patient clinical image	Sex/Age	Relevant medical history for diagnosis	Clinical Diagnosis	Histological Diagnosis	Diagnostic Agreement with AI	AI Diagnosis
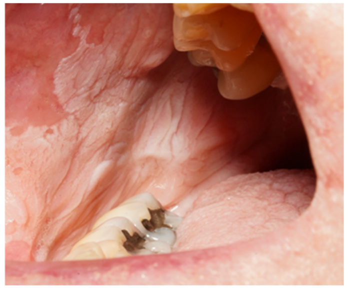	F/57	Bilateral Lesions	PVL	PVL	YES	
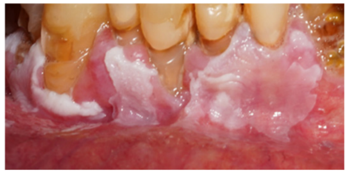	F/62	Burning<br>Bilateral Lesions<br>4 years Follow-up	PVL	PVL	YES	
**ACTINIC CHEILITIS**						
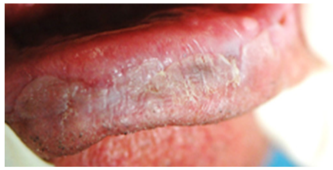	M/78	1 years Follow-up	AC	AC	YES	
**ORAL LICHENOID LESION**						
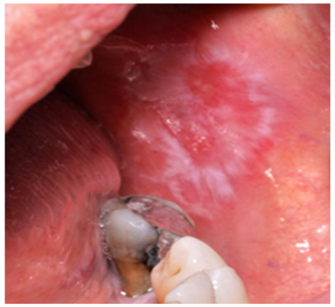	M/72	Burning<br>Pain<br>Monolateral lesion<br>Amalgama restorations	OLL	OLL	YES	

Patient clinical image	Sex/Age	Relevant medical history for diagnosis	Clinical Diagnosis	Histological Diagnosis	Diagnostic Agreement with AI	AI Diagnosis
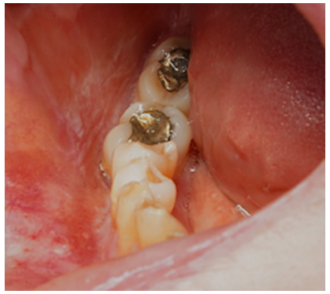	F/55	Burning<br>Pain<br>Mono-lateral Lesion<br>Amalgam restorations<br>1 years Follow-up	OLL	OLL	YES	
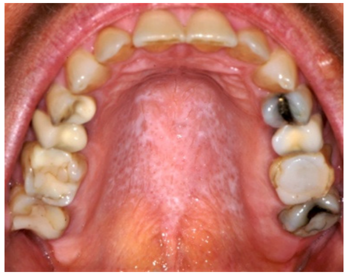	M/51	Amalgam restorations<br>Burning	OLL	OLL	YES	
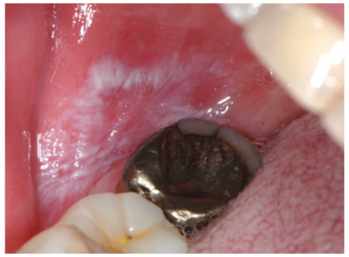	F/71	Amalgam restorations<br>Mono-lateral Lesion	OLL	OLL	YES	
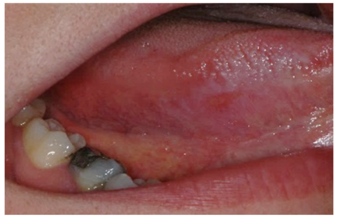	M/63	Amalgam restorations<br>Mono-lateral Lesion<br>2 years Follow-up	OLL	OLL	YES	

Patient clinical image	Sex/Age	Relevant medical history for diagnosis	Clinical Diagnosis	Histological Diagnosis	Diagnostic Agreement with AI	AI Diagnosis
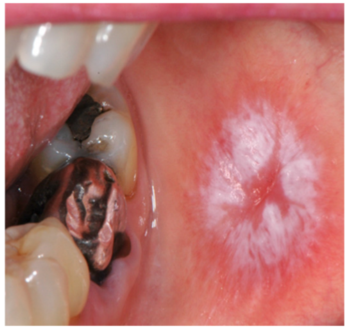	F/77	Amalgam restorations<br>Mono-lateral Lesion<br>Burning	OLL	OLL	YES	
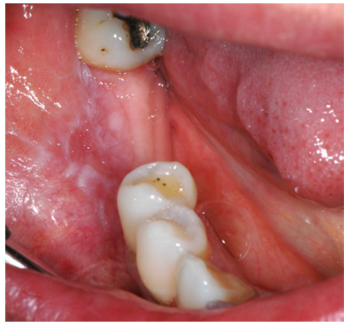	M/70	Amalgam restorations<br>3 years Follow-up<br>Mono-lateral Lesion	OLL	OLL	YES	
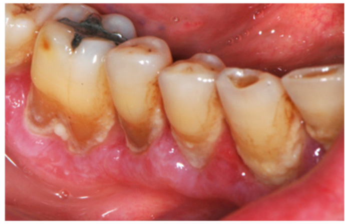	M/64	Amalgam restorations<br>Pain<br>Burning<br>Mono-lateral Lesion	OLL	OLL	YES	

Patient clinical image	Sex/Age	Relevant medical history for diagnosis	Clinical Diagnosis	Histological Diagnosis	Diagnostic Agreement with AI	AI Diagnosis
**GRAFT VERSUS HOST DISEASE**						
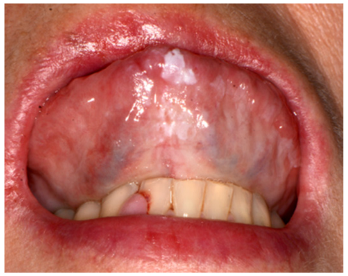	F/46	Bone Marrow Transplant<br>Burning<br>Pain	GVHD	GVHD	YES	
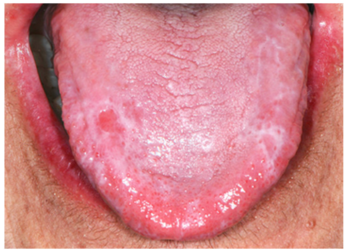	F/55	Bone Marrow Transplant<br>3 years Follow-up	GVHD	GVHD	YES	
**SYSTEMIC LUPUS ERYTHEMATOSUS**						
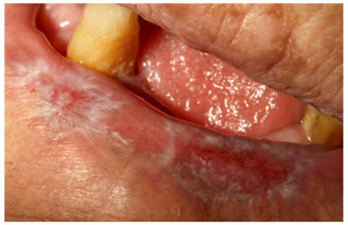	F/82	Burning<br>Pain<br>6 years Follow-up	SLE	SLE	YES	
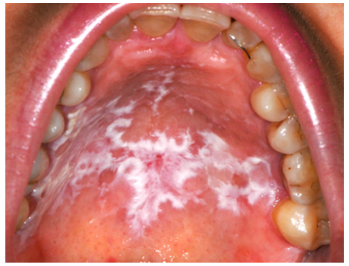	M/32	Burning<br>Pain	SLE	SLE	YES	

Patient clinical image	Sex/Age	Relevant medical history for diagnosis	Clinical Diagnosis	Histological Diagnosis	Diagnostic Agreement with AI	AI Diagnosis
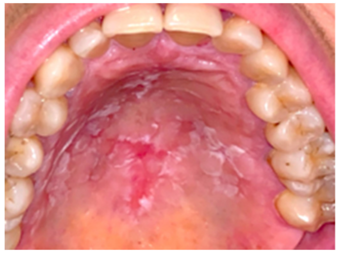	F/77	3 years Follow-up<br>Pain	SLE	SLE	YES	
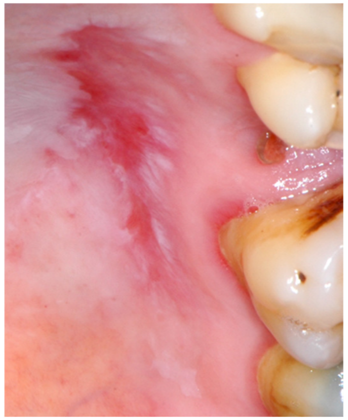	M/68	Burning<br>Pain<br>2 years Follow-up	SLE	SLE	YES	
**PALATE KERATOSIS ASSOCIATED WITH SMOKING**						
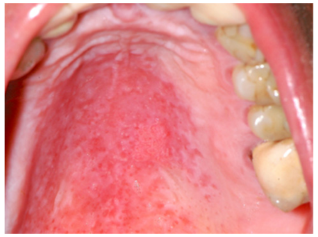	F/62	Smoker (30 cpd)<br>Drinker of 3 U/die<br>Burning	PKAS	PKAS	YES	

Patient clinical image	Sex/Age	Relevant medical history for diagnosis	Clinical Diagnosis	Histological Diagnosis	Diagnostic Agreement with AI	AI Diagnosis
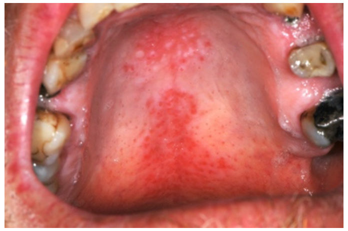	M/65	Smoker (40 cpd)<br>Burning<br>2 years Follow-up	PKAS	PKAS	YES	
**ORAL VERRUCOUS CELL CARCINOMA**						
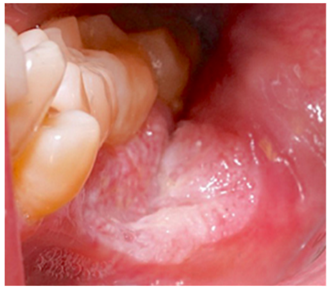	M/55	Chronic persistent lesion (8 months)	OVC	OVC	YES	
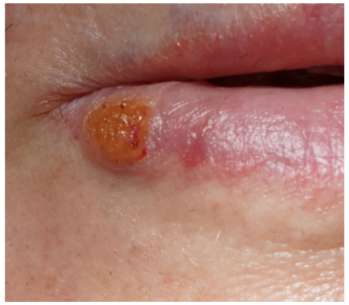	F/74	Burning<br>Pruritus<br>Erythema	OVC	OVC	YES	
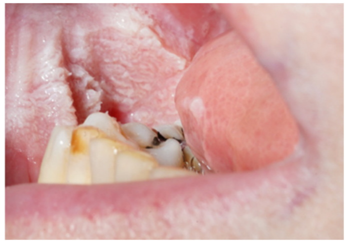	F/74	Burning<br>Pain<br>Bilateral Buccal and gingival lesions<br>9 years Follow-up	NHOL, OVC	OVC	YES	

Patient clinical image	Sex/Age	Relevant medical history for diagnosis	Clinical Diagnosis	Histological Diagnosis	Diagnostic Agreement with AI	AI Diagnosis
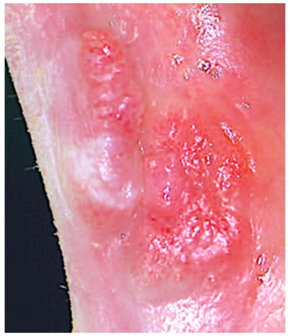	M/55	Smoker<br>(15 cpd)<br>Drinker 2 U/die<br>Non-Homogeneous Lesion<br>Retro-commissural Lesion	OVC	OVC	YES	
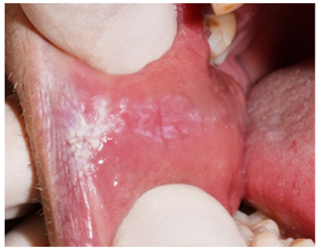	M/37	Smoker<br>(40 cpd)<br>Drinker of 1U/die<br>Exophytic Non-Homogeneous Lesion	NHOL	OVC	NO	NHOL
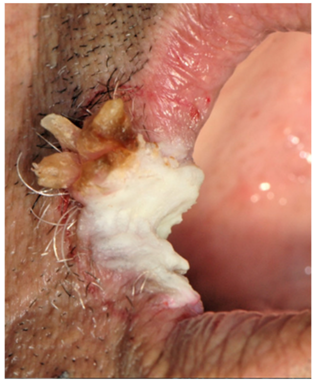	M/80	Smoker (10 cpd)<br>Pain<br>Exophytic Verruciform Lesion	OVC	OVC	YES	

Patient clinical image	Sex/Age	Relevant medical history for diagnosis	Clinical Diagnosis	Histological Diagnosis	Diagnostic Agreement with AI	AI Diagnosis
			**ORAL SQUAMOUS CELL CARCINOMA**			
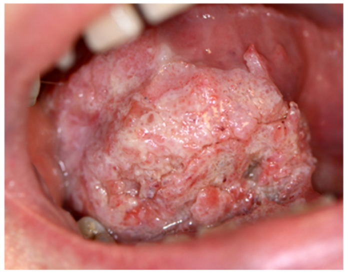	M/43	No identifiable risk factors<br>Persistent Lesion (6 months)<br>Lateral Tongue Ulcerated Lesion<br>Dysphagia	OSCC	OSCC	YES	
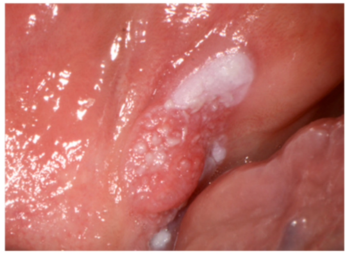	F/60	4 years Follow-up<br>Chronic Trauma<br>Smoker<br>(20 cpd)<br>Lesion of the Anterior Tonsillar Pillar	OSCC	OSCC	YES	
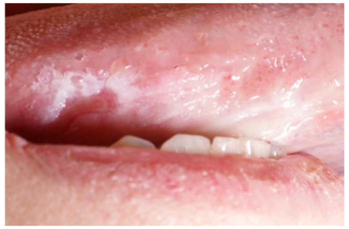	F/35	Persistent Ulcer<br>Pain<br>Burning<br>3 years Follow-up<br>Lateral tongue lesion	NHOL	OSCC	NO	NHOL
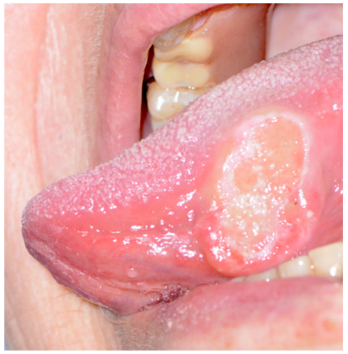	F/66	Repetitive trauma<br>Persistent Lesion (1 years)<br>Pain<br>Ill-fitting Dental Crowns	OSCC	OSCC	YES	

Patient clinical image	Sex/Age	Relevant medical history for diagnosis	Clinical Diagnosis	Histological Diagnosis	Diagnostic Agreement with AI	AI Diagnosis
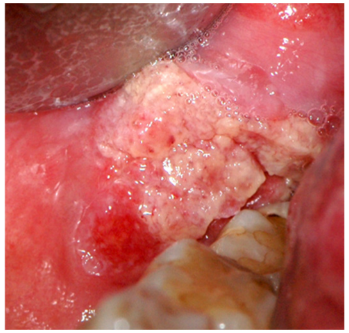	M/69	Bruxism<br>Persistent lesion (1-2 years)<br>Smoker (20 cpd)<br>Retromolar trigone	OSCC	OSCC	YES	
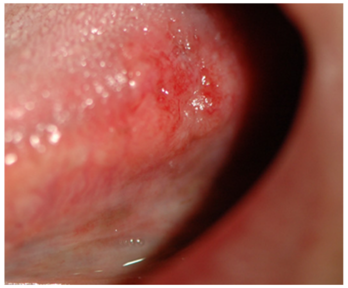	M/40	No risk factors<br>Lateral tongue lesion	OSCC	OSCC	YES	
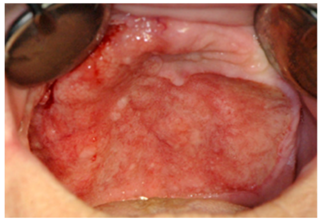	F/88	Removable denture wearer<br>Pain<br>Smoker (20 cpd)	EP	OSCC	YES	
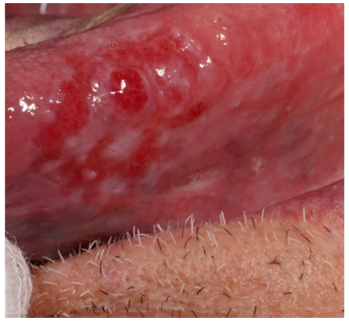	M/66	HCV+<br>Burning<br>Pain<br>10 years Follow-up<br>Lateral Tongue Non-Homogeneous Lesion	EP	High-grade OED/CIS	YES	

Patient clinical image	Sex/Age	Relevant medical history for diagnosis	Clinical Diagnosis	Histological Diagnosis	Diagnostic Agreement with AI	AI Diagnosis
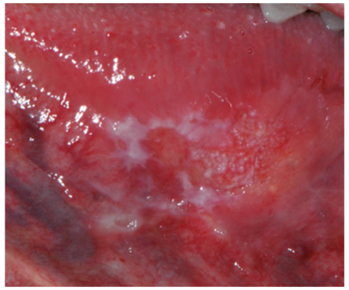	F/70	Smoker (10 cpd)<br>Drinker of 2 U/die<br>Burning<br>Lateral Tongue Non-Homogeneous Lesion	NHOL	OSCC	YES	
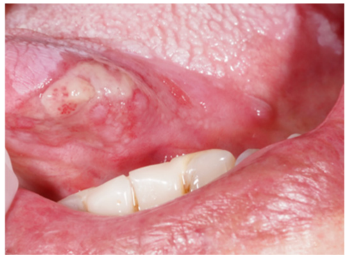	F/76	Pain<br>Lateral Tongue Non-Homogeneous Lesion	OSCC	High-grade OED/CIS	YES	
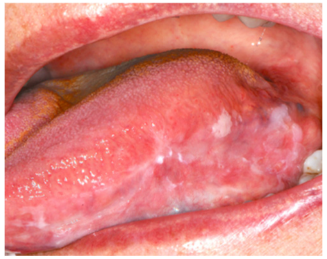	F/78	Pain<br>Persistent ulcer<br>Lateral Tongue Non-Homogeneous Lesion<br>3 years Follow-up	NHOL	OSCC	YES	
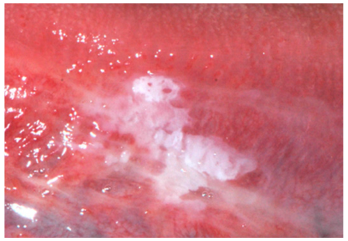	F/56	Burning<br>Xerostomia<br>Smoker (20 cpd)<br>Drinker 1 U/die<br>Lateral Tongue Non-Homogeneous Lesion	NHOL	OSCC	YES	

Patient clinical image	Sex/Age	Relevant medical history for diagnosis	Clinical Diagnosis	Histological Diagnosis	Diagnostic Agreement with AI	AI Diagnosis
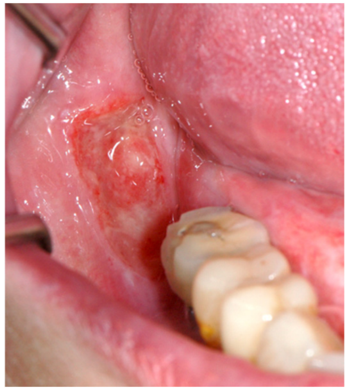	F/55	Burning<br>Persistent lesion (6 months)<br>Retromolar trigone ulcer	OSCC	OSCC	YES	
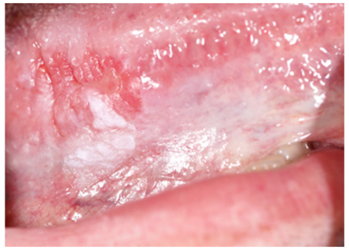	F/50	Pain<br>Smoker (20 cpd)<br>Lateral tongue<br>Non-Homogeneous Lesion<br>3 years Follow-up	OSCC	OSCC	YES	
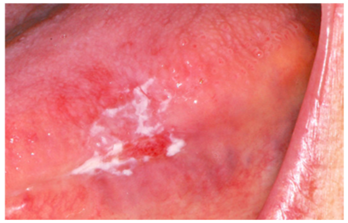	M/58	Pain<br>Lateral tongue<br>Non-Homogeneous Lesion<br>-Smoker (10 cpd)	OSCC	OSCC	YES	

Patient clinical image	Sex/Age	Relevant medical history for diagnosis	Clinical Diagnosis	Histological Diagnosis	Diagnostic Agreement with AI	AI Diagnosis
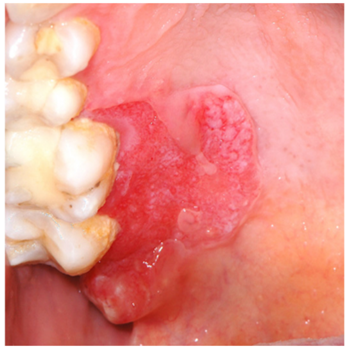	F/59	Burning<br>Persistent Lesion (1 years)<br>Pain	OSCC	OSCC	NO	EP
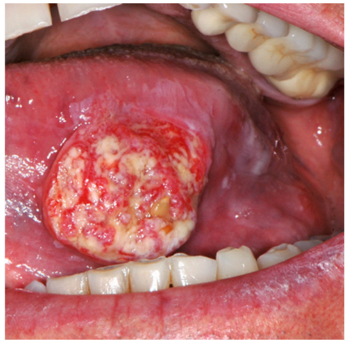	F/77	Smoker (10 cpd)<br>Lateral tongue ulcerated lesion<br>Pain	OSCC	OSCC	YES	
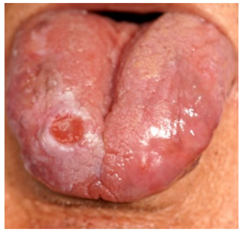	M/92	Persistent Non-Homogeneous Lesion<br>Burning<br>8 years Follow-up	OSCC	OSCC	YES	

Patient clinical image	Sex/Age	Relevant medical history for diagnosis	Clinical Diagnosis	Histological Diagnosis	Diagnostic Agreement with AI	AI Diagnosis
**BASAL CELL CARCINOMA**						
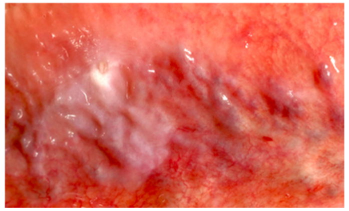	F/78	6 years Follow-up<br>Lateral tongue lesion	NHOL	nBCC	NO	NHOL
**UREMIC STOMATITIS**						
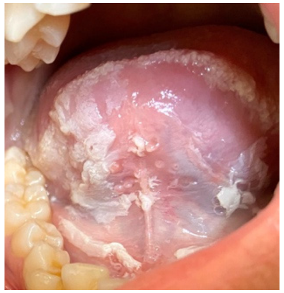	F/15	Acute onset<br>Pain<br>Halitosis<br>Laboratory Investigations: severe anemia (hemoglobin, 5.4 g/dL), markedly impaired renal function (serum creatinine, 13 mg/dL; glomerular filtration rate, 3.78 mL/min/1.73 m²)	US	US	YES	
**MUCOUS MEMBRANE PEMPHIGOID**						
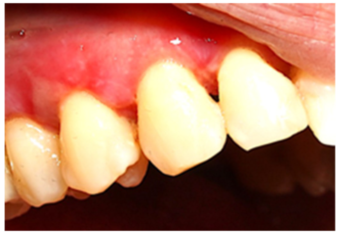	F/55	Burning<br>Pain<br>Epithelial detachment	MMP	MMP	YES	
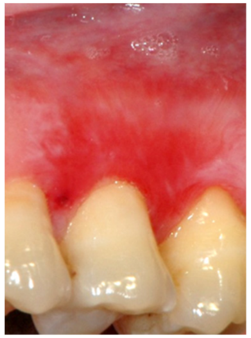	F/55	Burning<br>Bleeding while performing oral hygiene<br>Gums become ulcerated	MMP	MMP	YES	

Patient clinical image	Sex/Age	Relevant medical history for diagnosis	Clinical Diagnosis	Histological Diagnosis	Diagnostic Agreement with AI	AI Diagnosis
**VULGARIS PEMPHIGUS**						
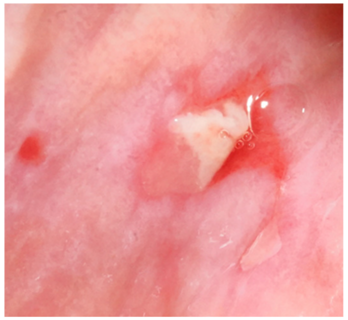	M/45	Erosive Lesions<br>Epithelial detachment<br>Burning<br>Pain<br>Bleeding<br>Cutaneous lesions	PV	PV	YES	
**CHRONIC HYPERPLASTIC CANDIDOSIS**						
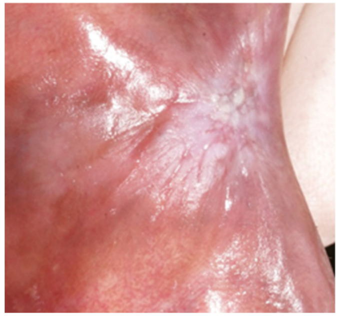	M/40	Smoker<br>(30 cpd)<br>Bilateral Lesions	CHC	CHC PAS+	YES	
**AMILOIDOSIS**						
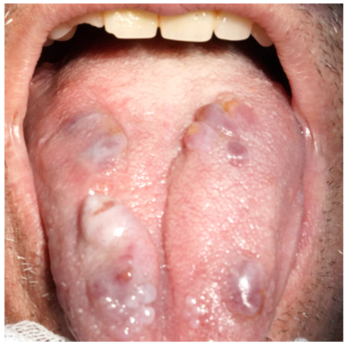	M/64	Multiple Myeloma<br>Multiple Nodular Lesion<br>Macroglossia	Amiloidosis	Amiloidosis	YES	

Patient clinical image	Sex/Age	Relevant medical history for diagnosis	Clinical Diagnosis	Histological Diagnosis	Diagnostic Agreement with AI	AI Diagnosis
			**PARANEOPLASTIC PEMPHIGUS**			
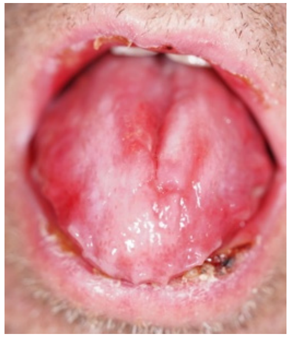	M/45	Non-Hodgkin Lymphoma<br>Burning<br>Pain<br>Dysfagia<br>Bullous Lesions	PNP	PNP	YES	
			**MORSICATIO BUCCARUM**			
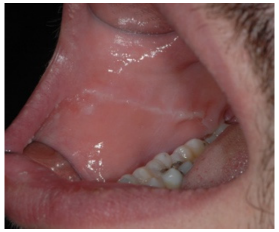	M/40		MB		YES	
			**COSMETIC REACTION LESION**			
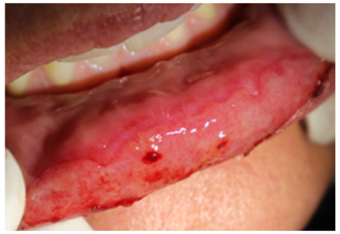	F/55	Use of cosmetic product<br>Burning	Cosmetic reaction		YES	

AC: Actinic Cheilitis; CHC: Chronic Hyperplastic Candidosis; CIS: Carcinoma in situ; cpd: cigarettes per day; EC: Erythematosus Candidiasis; EP: Eritroplakia; FK: Frictional Keratosis; GVHD: Graft Versus Host Disease; HOL: Homogeneus Oral Leucoplakia; MB: Morsicatio Buccarum; MMP: Mucous Membrane Pemphigoid; nBCC: nodular Basal Cell Carcinoma; NHOL: Non-Homogeneus Oral Leucoplakia; NSLCC: Non-Small Lung Cell Cancer; OED: Oral Epithelial Dysplasia; OLL: Oral Lichenoid Lesion; OLP: Oral Lichen Planus; OSCC: Oral Squamous Cell Carcinoma; OSP: Oral Squamous Papilloma; OVC: Oral Verrucous Carcinoma; PKARS: Palatal Keratosis Associated with Reverse Smoking; PNP: Paraneoplastic Pemphigus; PV: Pemphigus Vulgaris; PVL: Proliferative Verrucous Leucoplakia; SLE: Systemic Lupus Erythematosus; US: Uremic Stomatitis
